# The Scarlet Letter

**Published:** 1889-07-06

**Authors:** 


### "The Scarlet Letter."

While most hospitals are increasing their hold on the charitable public, and can boast of an increase, however slight, in their subscription list, the subscriptions to the London Lock Hospital are steadily going down. The reason of this is obvious. The disease treated at the Lock Hospital is the result of sin, and of a sin that is peculiarly repugnant to the best among us. Women are among the best friends of hospitals, and many good women look upon a lock hospital simply as an integral part of a vice which all their instincts lead them to regard with abhorrence. The repeal of the Contagious Diseases Acts was obtained by the general feeling that their existence implied a national approval of impurity; many people feel that to contribute to an institution which tries to cure the victims of that sin implies an approval, or at least a toleration, of the sin itself.

To such ideas a visit to the London Lock Hospital in Harrow-road would be the best antidote; but since that is impracticable to many of our readers we would ask them to bear with us while we say a few words with regard to those of our sisters who bear in their body that "scarlet letter" which Hester Prynne wore on her gown. Sad as it is to think of, we know that many girls are brought up to a career of prostitution. They have been reared in surroundings that make innocence of thought impossible; they have been trained to sin for bread; they know no other means of gaining a living. Others fall once through passion, and go on in evil simply from despair. They are ashamed to go back to their homes; they know or think they would not be received there, and so they go on the dreary downward way. Everything is made hard to a woman who has once erred. How few will give them employment, or, having done so, will make allowance for the morbidly sensitive nature they so often—the best of them—possess, or be patient with the restless passionate temperament which, while it led to their fall, is an integral part of their constitution, and can only be controlled, not eradicated. Many people seem to think that because the way of transgressors is hard that of penitents should be hard also, and treat women whose moral nature has been proved weak with a severity that would drive stronger and more patient natures to rebellion.

To these comes God's messenger—disease. Admitted to a hospital, they come in contact for the first time with women who are both pure and gentle; with men who, even while treating the diseases that result from sin, preserve a reverence for their womanhood. The work of rescue goes on hand-in-hand with the work of cure; new ideas of life, new possibilities of work, arise in their minds. We are not drawing a fancy picture in saying this; it is matter of fact that rather more than a quarter of the patients admitted into the hospital in Harrow-road renounce their evil lives. It is a rule of the hospital that as soon as a girl expresses a wish to break away from her past career she is removed to another ward, kept for hopeful cases, and from there, when recovered, drafted into the home, or asylum, connected with the hospital. Here they are taught sewing, laundry-work, housemaid's duties, and cooking; so that, when they leave it after a year's probation, they have a knowledge of honest means of earning a living. That they are well taught may be inferred from the fact that ladies who have once had a servant from the home usually go back for another. Often, unfortunately, it is difficult to obtain situations for them; so few will give them a chance of doing right.

So far, we have spoken of fallen women; but among the patients at the Lock Hospital are innocent ones also. Wives suffer for their husband's sins, and children are born to an inheritance of disease. Cases of both kinds are at the hospital now. But even for the others we would plead that to help an institution which cures and tries to redeem these unhappy women is a practical way of bidding them "Go, and sin no more."

Contributions should be sent to the secretary, Mr. Algernon C. Coote, Lock Hospital, Harrow-road, W.

